# Hair-Growth-Promoting Effects of the Fish Collagen Peptide in Human Dermal Papilla Cells and C57BL/6 Mice Modulating Wnt/β-Catenin and BMP Signaling Pathways

**DOI:** 10.3390/ijms231911904

**Published:** 2022-10-07

**Authors:** Su Bin Hwang, Hyeon Ju Park, Bog-Hieu Lee

**Affiliations:** Department of Food and Nutrition, Chung-Ang University, Anseong 17546, Gyeonggi, Korea

**Keywords:** fish collagen peptides, hair growth factors, Wnt/β-catenin pathways, BMP signaling pathways, human dermal papilla cells, C57BL/6 mice

## Abstract

Fish-derived collagen has recently emerged as an alternative collagen source with bioactive properties, including the enhancement of hair and skin health. It is also cost-effective and has high bioavailability, in addition to having fewer side-effects compared to collagen from porcine skin or bovine skin. Collagen peptides (CPs) extracted from the scales of Mozambique tilapia (*Oreochromis mossambicus*) reportedly promote hair and skin health. This study sought to evaluate the effects of CPs on hair growth using in vitro and in vivo models. CP significantly enhanced hair regrowth and the proliferation of human dermal papilla cells (hDPCs) in vitro. CP was orally administered to C57BL/6 mice for 6 weeks to confirm the hair-growth-promoting effects. The mice were divided into four groups: negative control (distilled water), positive control (1 mg/kg of finasteride), CP500 (500 mg/kg of CP), and CP1000 (1000 mg/kg of CP). CP treatment significantly enhanced the proliferation of hDPCs compared to 0.2 μM finasteride, in addition to enhancing hair regrowth. Particularly, CP1000 treatment achieved a hair-growth index similar to that of the PC. In H&E staining, the CP groups exhibited a high A/T ratio. Furthermore, CP increased the expression of hair growth factors (IGF-1, VEGF, krt27, Gprc5d, and Ki67) and decreased the growth inhibitory factor (TGF-β1). Furthermore, CP significantly upregulated the Wnt/β-catenin pathways and downregulated the BMP pathways. Therefore, these results indicate that CP could be used as food supplements and nutraceuticals for hair loss prevention as well as hair regrowth during alopecia.

## 1. Introduction

Hair growth involves the growth and differentiation of hair follicles comprising dermal papilla cells and epithelial cells [1]. The life cycle of a hair follicle is divided into three phases: anagen (growth), catagen (regression), and telogen (rest) [2]. Normally, hair follicles are contracted after the anagen phase and the hair shaft falls out during the catagen or telogen phase. As the follicles begin a new anagen phase, they grow back to their original size and produce new hair of a normal thickness [3,4]. An increase in the number and size of hair follicles is considered an indicator of the telogen-to-anagen transition, which can be observed by monitoring the deep subcutis during the anagen phase [5].

Promoting anagen elongation and the telogen-to-anagen transition is crucial for maintaining healthy hair. In turn, the hair cycle transition is controlled by various growth stimulatory or inhibitory factors [6,7,8,9], many of which are expressed in the hair follicles. Ki67, vascular endothelial growth factor (VEGF), insulin-like growth factor (IGF-1), G-protein coupled receptor family C group 5 member D (Gprc5d), and keratin 27 (Krt27) act as hair growth stimulators, whereas transforming growth factor beta-1 (TGF-β1) is a hair growth suppressor [10]. Ki67 is well known marker for hair follicle proliferation and morphogenesis [11,12]. IGF-1 and VEGF prolong the duration of the anagen phase, whereas TGF-β1 induces the catagen phase [4,13]. Gpr5cd is highly expressed in the late growth phase (anagen) but is not expressed in the resting phase (telogen) [14,15]. Krt27 is a hair-follicle-specific epithelial keratin that is mainly involved in hair follicle morphogenesis and is known to be upregulated during keratinocyte differentiation [15,16].

Wnt/β-catenin signaling was recently found to play a crucial role during developmental hair follicle induction [4]. Specifically, Wnt10b is prominently upregulated in the hair follicle epithelium at the onset of the anagen phase [17,18,19,20,21], and β-catenin is involved in hair follicle development and is an essential mediator of the differentiation of stem cells into hair follicles [19,22]. Bone morphogenetic proteins (BMPs) belong to the TGF-β family and plays an essential role in controlling the initiation of the hair follicle growth phase by stopping cell proliferation and differentiation during epidermal development [23,24,25]. Specifically, BMP4 and BMP6 arrest the follicle during the telogen phase [4,23,24,26].

Low-molecular-weight collagen peptides (CPs) supplementation has recently garnered increasing attention due to its potential health benefits including hair/skin health [27,28,29,30,31,32,33]. Particularly, fish-derived CPs possess some advantages over conventional collagen sources, including a higher collagen content, environmental friendliness, low toxicity, low inflammatory response, greater absorbability due to its low molecular weight, less religious and ethical constraints, and less regulatory and quality control problems [34,35]. Patients suffering from hair loss often complain about scalp pruritus, scalp scaling, scalp irritation, undesirable hair texture, and the need to apply medication twice a day. However, research on hair growth therapies has mostly focused on direct topical treatments, whereas oral treatments have remained largely unexplored [36]. Among fish CPs, CP extracted from the scales of the Mozambique tilapia (*Oreochromis mossambicus*) has been reported to possess several bioactive properties such as hair cell proliferation [37], antioxidant [38], and wound healing effects [39]. In previous studies, bioactive CPs have been reported to enhance hair thickness and follicle cell proliferation, in addition to improving hair dryness and dullness [29,30,40].

To the best of our knowledge, the effects of oral administration of CP from tilapia on hair growth and its action mechanism have not been comprehensively characterized. Considering its various therapeutic properties, cost-effectiveness, high bioavailability, and mild side-effects, fish CP could be used as a supplement to promote hair growth. Moreover, fish CP can be taken orally, thus providing a more convenient route of administration compared to topical treatment. Therefore, our study sought to evaluate the effects of oral fish CP supplementation on hair growth and its related action mechanisms.

## 2. Results

### 2.1. Effect of CP on Cell Proliferation of Human Dermal Papilla Cell

**Figure 1:**
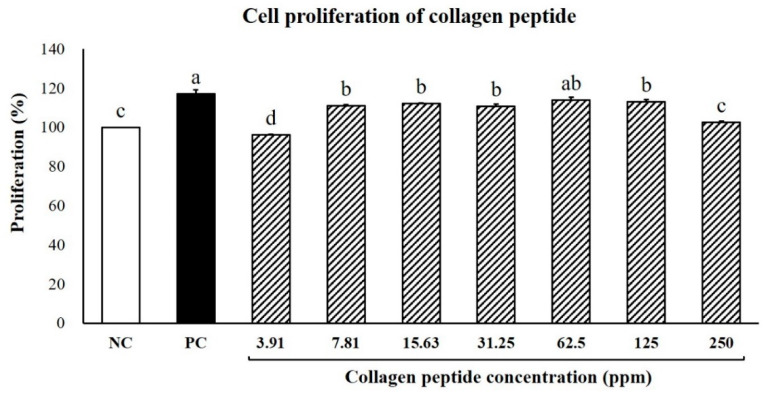
Effects of CP on hDPCs proliferation. NC: negative control (DMEM); PC: positive control (0.2 μM finasteride); CP: collagen peptides (3.91, 7.81, 31.25, 62.5, 125, and 250 ppm). Values are expressed as the mean ± SE. Values with different superscript letters differ significantly at *p* < 0.05.

To evaluate the safety of CP administration, the proliferation rate of hDPCs was evaluated at various concentrations of CP (Figure 1). The cell proliferation rate of the NC group was adjusted to 100 as a reference and all other treatments were reported as relative values. The CP 62.5 ppm group exhibited the highest hDPCs proliferation rate (113.98 ± 1.44%). More importantly, this value was not significantly different from the PC group. The CP 7.81–125 ppm groups exhibited similar cell proliferation rates, all of which were higher than that of the NC group (*p* < 0.05). CP showed a higher cell proliferation than NC at all concentrations except 3.91 ppm.

### 2.2. Hair Growth on Dorsal Skin after Oral Administration of CP

**Figure 2:**
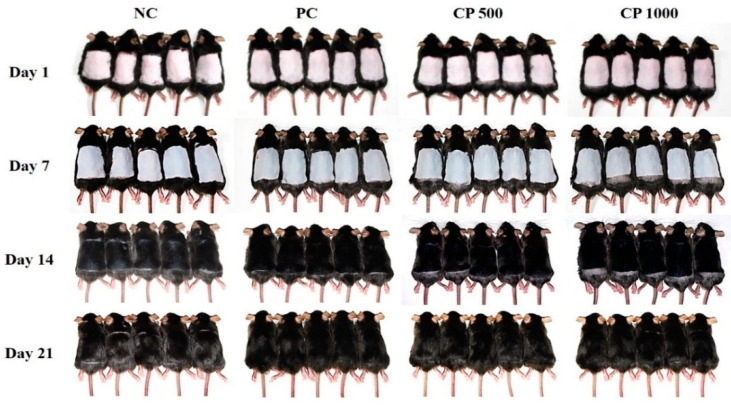
Effect of CP on anagen phase induction in C57BL/6 mice after depilation. Before and after depilation, distilled water (vehicle), finasteride (1 mg/kg in distilled water), and CP (500 and 1000 mg/kg in distilled water) were orally administrated to each group for 6 weeks. Dorsal skins were photographed on days 1, 7, 14, and 21 after depilation. NC: negative control (distilled water); PC: positive control (finasteride); CP500: collagen peptide, 500 mg/kg; CP1000: collagen peptide, 1000 mg/kg.

Photographs were taken at 7-day intervals to visually confirm the hair growth pattern of the mouse dorsal skin (Figure 2). On the first day of hair removal, it was confirmed that all groups had pink skin. After 7 days, the dorsal skin began to appear gray in all groups. Particularly, the NC group was very pale gray, whereas the PC and CP groups were a darker shade of gray compared to the NC group. Moreover, both CP500 and CP1000 mice showed a darker dorsal skin color than the PC group. After 14 days, hair growth was observed on the dorsal skin of all mice. When comparing the groups, the NC group had shorter hair and the dorsal skin was partially visible, whereas both the dorsal skin of the PC and CP mice appeared fully covered. At 21 days, all groups exhibited full hair growth. Regarding the hair growth pattern, the CP1000 group (i.e., the group treated with the highest CP concentration) exhibited a darker dorsal skin color, confirming that CP promotes hair growth by inducing the anagen phase.

### 2.3. Effects of CP on Hair Regrowth in C57BL/6 Mice

**Figure 3:**
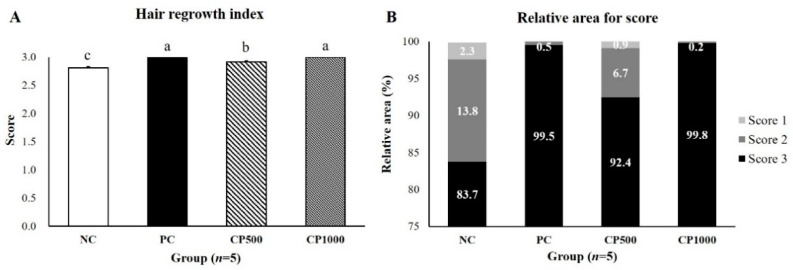
Effect of CP on hair regrowth score in depilation-induced C57BL/6 mice. (**A**) Hair regrowth score and (**B**) relative area for a score. The hair regrowth score was calculated by the skin dorsal photo on day 14. Differences were calculated by measuring the size of the complete black area (black skin) using the Image J program. Values with different superscript letters are significantly different (*p* < 0.05). The results are reported as the mean ± SE (*n* = 5/group). NC: negative control (distilled water); PC: positive control (finasteride, 1 mg/kg); CP500: collagen peptide, 500 mg/kg; CP1000: collagen peptide, 1000 mg/kg.

The hair regrowth score and area of all groups at 14 days were calculated using the ImageJ software to investigate the effect of CP intake on hair growth in mice, including the hair regrowth score and area of all groups at 14 days (Figure 3A). The PC and CP groups exhibited a significantly higher hair regrowth score than the NC group. Particularly, all the hairs on the back skin of the CP1000 mice had already grown with a score of 3 (i.e., the highest hair regrowth score) on the 14th day. The hair regrowth score of the PC group was 2.99 ± 0.00, showing no significant difference from the CP1000 group. Moreover, the CP500 group showed a higher hair regrowth score than the NC group (*p* < 0.05).

Furthermore, after measuring the dorsal area of each mouse according to the hair regrowth score, the NC group exhibited the largest area with a score of 1 (*p* < 0.05). The NC group also exhibited the largest score 2 areas, followed by the CP500, PC, and CP1000 groups. The PC and CP1000 groups showed no significant differences. The score 3 areas, which were indicative of full hair growth, were higher in the PC group and CP1000 group than in the CP500 group and NC group (*p* < 0.05) (Figure 3B). Therefore, our findings demonstrated that CP increased the hair regrowth score and area of the mice in a concentration-dependent manner. Particularly, the hair-regrowth-promoting effect of the CP1000 treatment was similar to that of finasteride (i.e., the positive control; PC), thus confirming that CP effectively promoted hair regrowth.

### 2.4. Effects of CP on Hair Follicle Morphology

**Figure 4:**
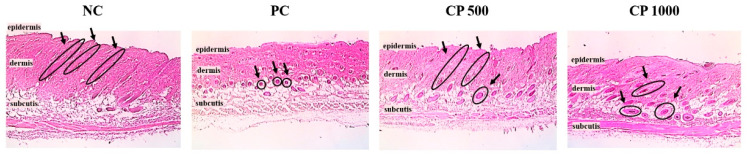
Histological effect of CP treatment. A/T ratio of the dorsal skin of C57BL/6 mice orally treated with CP (500 mg/kg and 1000 mg/kg) for 6 weeks. NC: negative control (distilled water), PC: positive control (finasteride, 1 mg/kg); CP500: collagen peptide, 500 mg/kg; CP1000: collagen peptide, 1000 mg/kg (hematoxylin & eosin stain, 100× magnification). The arrows represent the hair follicles.

Histological analysis of dorsal skin tissue, including the number of hair follicles and A/T ratio, was performed to investigate the effect of CP intake on the development of hair follicles and follicles in mice. After 3 weeks of oral administration, the longitudinal sections of dorsal skin tissue of mice were analyzed after staining with H&E (Figure 4).

Regarding the A/T ratio, the hair follicles of the NC group were generally located in the epidermis, and this group exhibited the lowest number of follicles compared to the other treatments (*p* < 0.05). In the PC and CP groups, hair follicles were located in the dermis, and the number of hair follicles was higher than in the NC group. Compared to the PC group, the CP group was located in the deep dermis close to the subcutis, and the hair follicles were larger and more mature. Particularly, the CP1000 group had larger and thicker hair follicles than the CP500 group.

**Figure 5:**
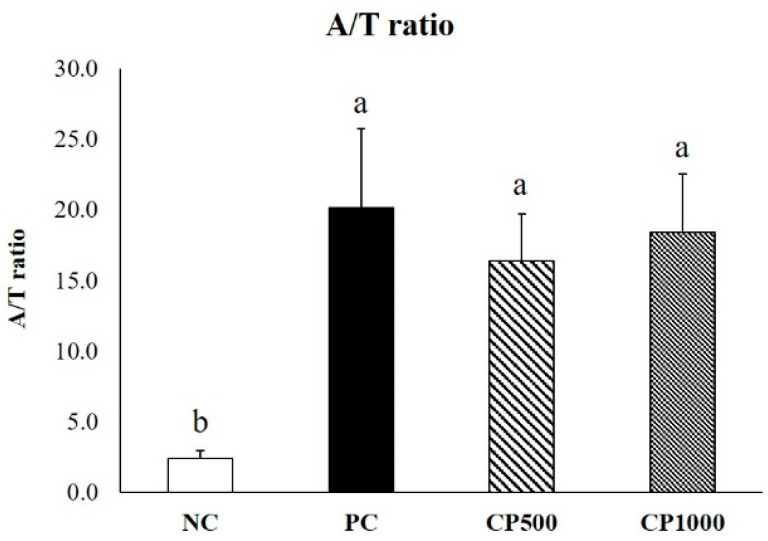
Effect of oral CP administration (500 mg/kg and 1000 mg/kg) on A/T ratio of C57BL/6 mice treated for 6 weeks. The follicles in the anagen phase exhibit a large and thick hair bulb located deep in the epidermis. The telogen phase features a small and flat hair bulb located in a shallow layer of the epidermis. The anagen and telogen phases were confirmed by a histopathologist. Values with different superscript letters are significantly different (*p* < 0.05). The results are the mean ± SE (*n* = 7/group). NC: negative control (distilled water), PC: positive control (finasteride, 1 mg/kg); CP500: collagen peptide, 500 mg/kg; CP1000: collagen peptide, 1000 mg/kg.

The A/T ratio of all groups was calculated by counting the number of telogen hair follicles (T) located in the epidermis and anagen hair follicles (A) located in the dermis and subcutis (Figure 5). The A/T ratio was significantly higher in all treatment groups compared to the NC group. The A/T ratio of the PC group was highest, followed by CP groups. Both CP500 and CP1000 groups had a greater number of follicles than the NC group. Compared to the PC group, the hair follicles of the CP group were located in the deeper dermis and the ratio of large and thick hair follicles was high. Therefore, our findings confirmed that CP increased the number of hair follicles, as well as the ratio of hair follicles in the anagen phase.

### 2.5. Effects of CP on Cytokine Expression of Mice Dorsal Tissues

**Figure 6:**
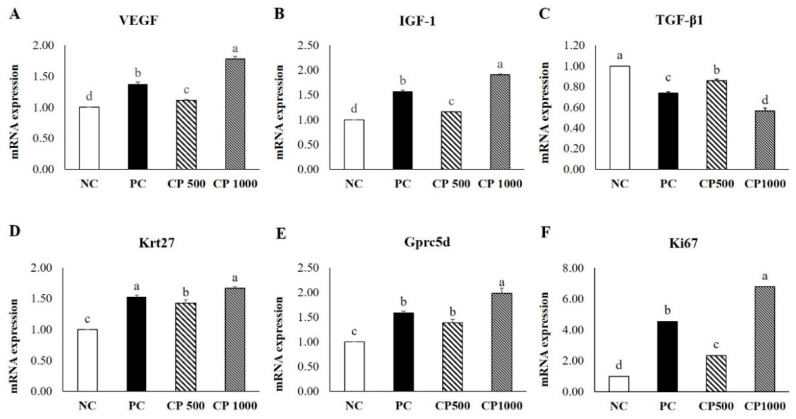
Results of RT-qPCR. Effect of hair regrowth on depilation-induced C57BL/6 mice orally treated with CP (500 mg/kg and 1000 mg/kg, respectively) for 6 weeks. (**A**) VEGF; (**B**) IGF; (**C**) TGF-β1; (**D**) Krt27; (**E**) Gprc5d; (**F**) Ki67. Values with different superscript letters are significantly different (*p* < 0.05). Results are the mean ± SE (*n* = 8/group). NC: negative control (distilled water), PC: positive control (finasteride, 1 mg/kg); CP500: collagen peptide, 500 mg/kg; CP1000: collagen peptide, 1000 mg/kg.

To confirm the effect of CP intake on hair growth in mice, the mRNA expression levels of hair-growth-promoting/inhibitory factors (VEGF, IGF-1, TGF-β1, Krt27, and Gprc5d) and hair bulb proliferation and growth-promoting factors (Ki67) were measured by RT-qPCR (Figure 6). The results were normalized to the NC group.

The CP1000 group exhibited a 1.3 times higher expression of VEGF than the PC group. The CP1000 group exhibited the highest mRNA expression of VEGF (*p* < 0.05), a factor that induces differentiation of hair root cells and increases the size and thickness of hair follicles through the improvement of blood circulation [41,42,43]. The CP500 group showed a significantly higher expression level of VEGF mRNA than the NC group. The mRNA expression level of IGF-1, a factor that promotes the proliferation of hair follicle epithelial cells and inhibits the transition from the anagen phase to the telogen phase [41,42,43], was highest in the CP1000 group, followed by the PC, CP500, and NC groups (*p* < 0.05). The mRNA expression of TGF-β1, which induces the anagen-to-telogen transition of hair follicles by triggering apoptosis of the hair follicles [44], was lower in the CP1000 group than the PC group (*p* < 0.05). Moreover, the mRNA expression of TGF-β1 was significantly lower in the CP500 group compared to the NC group.

The expression level of Gprc5d, a growth factor mainly expressed during the anagen phase of hair follicles [14,15], was highest in the CP1000 group (*p* < 0.05). Furthermore, the Gprc5d expression of the CP500 group was significantly higher than that of the NC group but was not significantly different from that of the PC group. CP was confirmed to inhibit the anagen-to-telogen transition by increasing the expression level of the hair-growth-promoting factor and decreasing the expression level of the hair growth inhibitory factor. The mRNA expression level of Krt27, a factor involved in hair follicle morphogenesis [15], was significantly higher in the PC and CP groups than in the NC group. Particularly, the CP1000 group showed a higher Krt27 expression level than the PC group (*p* < 0.05), and the CP500 group showed a similar level of expression to the PC group. Ki67 mRNA expression was highest in the CP1000 group (*p* < 0.05).

### 2.6. Effects of CP on Protein Expression of Mice Dorsal Tissues

**Figure 7:**
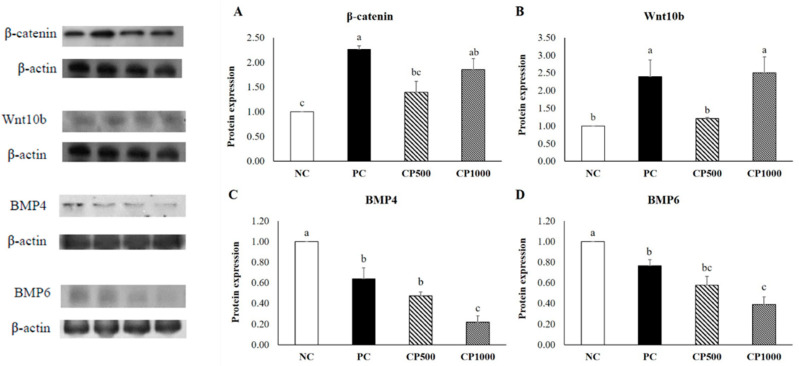
Results of Western blot analyses. Effect of CP on depilation-induced C57BL/6 mice. (**A**) β-catenin; (**B**) Wnt10b; (**C**) BMP4; (**D**) BMP6. Values with different superscript letters are significantly different (*p* < 0.05). The results are reported as mean ± SE (*n* = 8/group). NC: negative control (distilled water), PC: positive control (finasteride, 1 mg/kg); CP500: collagen peptide, 500 mg/kg; CP1000: collagen peptide, 1000 mg/kg.

To determine the effects of CP on relevant signaling pathways, the expression levels of representative proteins involved in the hair-growth-related Wnt/β-catenin signaling pathway and the hair-inhibition-related BMP signaling pathway were measured (Figure 7). Protein expression was then calculated relative to the NC group. The CP group exhibited high Wnt10b and β-catenin expression levels. Particularly, the CP1000 group exhibited expression levels similar to those of the PC group. These findings suggest that CP activates hair-growth-related signaling pathways. BMP4 and BMP6 protein expression was evaluated to assess the effect of CP on the BMP signaling pathway. Both the CP500 and CP1000 groups showed low protein expression compared to the NC and even the PC. Particularly, CP1000 showed the lowest expression of both the BMP4 and BMP6 proteins, suggesting that CP inhibits hair-inhibition-related signaling pathways.

## 3. Discussion

The hair growth cycle is regulated by the interaction between hair follicle dermal cells and epithelial cells. As with hair follicle morphogenesis, proliferation and differentiation of epithelial cells and dermal papilla cells (DPCs) located at the base of the hair follicle are essential for hair follicle growth [45,46]. According to previous studies, stimulating the proliferation of DPCs has been shown to promote hair growth by inducing the anagen phase of the hair cycle [46]. In our study, CP showed a high rate of hDPCs proliferation at all concentrations. Previous studies have reported that *Geranium sibiricum* extract [11] and *Miscanthus sinensis* var. *purpurascens* flower extract [47] promote hair growth. However, the proliferation rate of hDPCs treated with *Geranium sibiricum* and *Miscanthus sinensis var. purpurascens* flower extract was drastically reduced at high concentrations of 156.3 ppm and 62.5 ppm, respectively, meaning that these extracts are toxic to hDPCs at high concentrations. In contrast, cell proliferation rates remained high even at a high CP concentration of 250 ppm. Therefore, fish CP is considered safe within a 3.91–250 ppm range and effectively induces the anagen phase of hair follicles by promoting hDPCs proliferation.

Hair growth could be regulated by modulating the hair cycle, such as by prolonging the anagen phase and promoting the telogen-to-anagen transition [46]. The C57BL/6 mice possess melanocytes only in the hair follicles, and therefore, melanin synthesis coincides with the hair growth cycle. Thus, the hair growth cycle can be easily characterized by simply monitoring the transition of the skin color from pink (no hair) to black (fully grown hair) [48]. During the telogen phase, the dorsal skin color of the mouse was pink, but shifted to gray and black over the course of a few weeks [49]. The hair regrowth score of mice orally treated with CP was significantly higher than that of untreated mice. The black, full-grown areas were also wider in the CP-treated mice than in the controls. These findings, thus, confirm that oral intake of fish CP promotes the telogen-to-anagen transition. The size and location of hair follicles vary according to each cycle of the anagen phase, catagen phase, and telogen phase [49]. In the anagen phase, the dermal papilla and hair bulb are large and located in the deep subcutaneous tissue. In the telogen phase, however, the dermal papilla is small and the hair follicle is located in the dermis close to the epidermis [49,50]. The hair follicles in the NC group were located in the epidermis, whereas hair follicles in the CP groups were mostly located deep in the subcutaneous tissue. These observations suggest that fish CP induces the anagen phase.

The hair cycle is regulated by specific cytokines, which affect the growth of mesenchymal-cell-derived DPCs. In turn, the affected DPCs release factors that either inhibit or promote the growth of follicular epithelial cells [41,45]. IGF-1, which is widely known as a hair-growth-promoting factor, promotes the proliferation of hair follicle epithelial cells and inhibits the anagen-to-catagen transition of hair follicles. Additionally, VEGF, a known hair growth factor, has been shown to increase follicle size and hair thickness by inducing the differentiation of hair root cells through the improvement of blood circulation [41,42,43]. Conversely, TGF-β1, known as a hair growth inhibitory factor, causes hair loss by inducing apoptosis, increasing the number of telogen hair follicles, and promoting entry into the catagen phase [13,20,44,51,52]. Our findings confirmed that the mRNA expressions of IGF-1 and VEGF of the CP-treated groups were upregulated compared to the PC group, whereas TGF-β1 was downregulated. In previous studies, increased mRNA levels of IGF-1 and VEGF, and decreased levels of TGF-β1 were associated with hair-growth-promoting effects [11,53]. Many studies have evaluated the effects of several compounds in a variety of presentations (e.g., ampoules, sprays, and ointments) on hair growth. However, little is known regarding the effectiveness of orally administered compounds for the treatment of hair loss. Previous studies have demonstrated that oral CP administration can improve microcirculation and increase blood supply, which could have an effect on the metabolism of the hair cells and subsequently lead to increased hair thickness [29]. Here, we confirmed that oral fish CP administration promoted the expression of hair-growth-promoting factors while suppressing the expression of hair growth inhibitory factors, thereby affecting hair growth.

Most studies on hair loss treatment have mainly targeted topical treatments rather than oral supplementation. A few studies had preliminarily explored the effects of oral CP intake on hair growth. However, adequate nutrient intake is also critical to enhance the turnover rates and metabolic activity on hair follicles [4]. For example, keratin is a primary component of hair, and its synthesis requires adequate protein intake to maintain sufficient levels in the body. Furthermore, in a previous study, low levels of amino acids were associated with hair loss [2]. CP contains high levels of proline-hydroxyproline (Pro-Hyp) dipeptide, which enhances cell proliferation and hyaluronic acid synthesis in human dermal fibroblasts [54]. Oral administration of low-molecular-weight CP, which is a fish-derived collagen hydrolysate, regulated the synthesis of amino acids such as hyaluronic acid and hydroxyproline, thereby restoring skin damage caused by UVB irradiation and increasing skin moisture [45,46]. Moreover, fish CP promoted the recovery of collagen fibers and normal elastic fibers in the skin from degraded collagen and abnormal elastic fibers caused by UVB irradiation [55]. Another study suggested that an oral composite supplement containing fish derived-CP and ornithine improves skin conditions by increasing plasma IGF-1 levels after 8 weeks of supplementation. Specifically, IGF-1 is known to activate cell growth both during epidermal and dermal skin development and maintenance [56].

The skin consists of the epidermis, dermis, and subcutaneous tissue. Collagen and elastin in the dermis maintain the structure of the skin and are responsible for its elasticity. The interactions between follicular keratinocytes and dermal papilla cells (specialized fibroblasts of the hair follicle) govern the formation of hair and hair cycling [10,56]. Moreover, given that keratin-associated proteins are produced during the anagen stage (growth stage) of the hair cycle, the upregulation of these proteins might be related to an induction of this stage [15]. Interestingly, skin and hair health are very closely related. For example, Pro-Hyp in keratinocytes in mouse skin upregulated hair-cycle-related genes such as Gprc5d and Krt27. Gprc5d is a member of the retinoic-acid-inducible gene-1 (RAIG-1) family. Gprc5d is highly expressed in the middle and late stage of the anagen phase of the hair cycle, but not in the telogen phase [14,15,57]. Krt27 is a hair-follicle-specific epithelial keratin that is upregulated during keratinocyte differentiation and is known to be mainly involved in hair follicle morphogenesis and the hair cycle [15,16,58,59]. Additionally, Ki67 is a well-known marker of hair follicle proliferation and morphogenesis [12]. In mice orally treated with CP, the mRNA expression levels of Gprc5d, Krt27, and Ki67 were all upregulated compared to the PC group. Upregulation of Krt27 induces the anagen stage, and keratin-associated protein (KRTAP) genes are mediated by several factors including BMP4 and Wnt10b. Moreover, as discussed above, cell proliferation is an essential driver of hair growth [10]. Induction of Ki67 suggests that the CP treatment activates cell proliferation of hDPCs by maintaining and stimulating the active phases of cell growth, as the Ki67 marker is exclusively expressed in the active phases of cell growth [11]. These results suggest that CP prolongs the anagen phase of hair follicles through the regulation of hair-cycle-related factors and is involved in hair follicle morphogenesis. Given the crucial role of hDPCs proliferation in the hair growth process, CP treatment likely maintains and stimulates the active phases of cell growth by inducing the expression of Ki67 only during the anagen phase [11]. These results suggest that fish CP prolongs the anagen phase of hair follicles through the regulation of hair-cycle-related factors and is involved in hair follicle morphogenesis.

Hair loss occurs through the modulation of several signaling pathways between follicular dermal cells and epithelial cells. Therefore, identifying the signaling pathways involved in hair follicle development is critical. Several signaling pathways, including Wnt/β-catenin and BMP, are involved in hair loss. Among them, activation of the Wnt/β-catenin signaling pathway determines the first skin signal [45]. When β-catenin accumulates in the cytoplasm by the Wnt protein and moves to the nucleus, it induces the anagen phase of hair follicles by the transcriptional co-activator of transcription factors belonging to the transcription factor T-cell factor/lymphoid enhancer factor (TCF/LEF) family. β-catenin plays an important role in the initiation, development, and growth of hair follicles [17,18,22]. Additionally, the Wnt paracrine signaling molecule acts early in the hair follicle formation process [45]. β-catenin, which plays a key role in the activation of the Wnt/β-catenin signaling pathway, is involved in hair follicle development and is an essential mediator of the differentiation of stem cells into hair follicles [19,22]. As the epidermis thickens, the dermis grows downward to form a follicle placode with dermal condensates. When the follicle placode is formed and stimulated, primary follicles are formed, which ultimately affects the length and thickness of the hair coat. The size of the placode and the size and number of dermal papilla cells are positively correlated with the thickness of the hair. β-catenin affects the formation and development of follicular placodes, ultimately increasing the length and thickness of the hair coat [60]. Additionally, Wnt10b is an essential factor in the initiation of the hair follicle cycle among Wnt proteins and is most highly expressed during the anagen phase. Wnt10b promotes hair follicle growth by promoting the proliferation of hair follicle cells via the Wnt signaling pathway [19,20,21]. Our findings indicated that the protein expression levels of β-catenin, which is involved in hair follicle differentiation and hair thickness, and Wnt10b, which induces the growth phase, were increased in CP-treated mice. Several studies have reached similar results through the regulation of the Wnt signaling pathway, and these changes in β-catenin and Wnt10b protein expression are thought to promote hair growth [19,61]. When β-catenin is downregulated, hair becomes shorter and thinner [19,22,62]. Oral CP administration increased β-catenin expression, thus preventing the hair from shortening and thinning, and the anagen phase was induced by increasing Wnt10b. In contrast to the Wnt/β-catenin signaling pathway, the BMP signaling pathway acts as an inhibitor of hair follicle formation [45]. BMPs are members of the TGF-β family, which play an essential role in controlling the initiation of the hair follicle anagen phase by stopping cell proliferation and differentiation during epidermal development [23,24,25]. Among them, BMP4 inhibits the anagen phase development of hair follicles by converting competent telogen follicles to refractory telogen follicles via the BMP-Smad pathway [23,26]. BMP4 also affects hair length and thickness by inhibiting stimulation of hair placode formation, thus reducing epidermal thickness and proliferation [63]. Furthermore, BMP6 is an important inhibitor of hair follicle regeneration. This inhibits the differentiation of hair follicle stem cells by activating the BMP signaling pathway and inhibiting the Wnt signaling pathway, leading to telogen arrest of the hair follicles [23,24]. Our findings confirmed that the protein expression levels of BMP4 and BMP6 decreased upon CP treatment. This suggests that fish CP intake can promote hair growth by inducing the anagen phase, delaying the telogen phase, and increasing the hair thickness. Therefore, fish CP induces the hair anagen phase by upregulating the Wnt/β-catenin signaling pathway involved in hair development and growth and downregulating the BMP signaling pathway, a hair growth inhibitory signal transduction pathway.

In conclusion, our findings demonstrated that oral fish CP administration enhanced the proliferation of hDPCs in vitro. CP promoted hair growth on dorsal skin tissue and induced a high A/T ratio in C57BL/6 mice. CP also significantly increased the mRNA expression of hair-growth-promoting factors such as Ki67, VEGF, IGF-1, Krt27, and Gprc5d, while downregulating the inhibition factor TGF-β1. In addition, CP upregulated Wnt/β-catenin and downregulated the BMP signaling pathway. Therefore, we concluded that oral administration of CP could induce the anagen phase and delay the telogen phase, promoting hair-growth factors and regulating hair cycles. Importantly, CP treatment promoted the expression of hair length and thickness-related factors, suppressed hair-inhibition factors, and modulated the expression of hair-cycle-related genes by regulating the Wnt/β-catenin and BMP signaling pathways. Further studies are needed to confirm these findings with human clinical studies. Considering the above research findings, fish CP could be used as a nutritional supplement for hair loss prevention and hair regrowth during hair loss.

## 4. Materials and Methods

### 4.1. Treatment and Cell Culture

The CP used in this study was a hydrolysate extracted from the scales of Mozambique tilapia (KER-30DR, manufactured by Nitta Gelatin India Limited Co., Cochin, India), and was obtained from Ju Yeong NS Co., Ltd. (Seoul, Korea). The CP was made by enzymatic hydrolysis and the licensed indicator was Gly-Pro-Val-Gly-Pro-Ser. The sample contained 128.79 mg/g of hydroxyproline, which is abundant in collagen (protein content is over 83%). Human dermal papilla cells (hDPCs) were obtained from Cell Engineering for Origin (CEFO BIO, Seoul, Korea). Dulbecco’s modified Eagle’s medium (DMEM) was prepared with 1% antibiotics and 10% fetal bovine serum (FBS). The cells were grown at 37 °C in a humidified 5% CO_2_ atmosphere.

### 4.2. CCK-8 Assay

The effects of CP on cell proliferation were evaluated via the CCK-8 assay. The hDPCs were seeded at a density of 3 × 10^3^ cells/well in a 96-well microplate for 24 h at 5% CO_2_ and 37 °C. CP was diluted to seven different concentrations (3.91, 7.81, 15.63, 31.52, 62.5, 125, and 250 ppm). These concentrations were commonly used in previous studies [11,47,61]. Afterward, 100 µL of each CP solution was added to each well and the cells were exposed for 24 h at 5% CO_2_ and 37 °C. DMEM, finasteride, and triton X-100 (Sigma) were used as the negative control (NC), positive control (PC), and blank, respectively. Next, 10 µL of CCK-8 solution (Sigma Aldrich, St. Louis, Mo., USA) was added to each well and the cells were incubated for 4 h at 37 °C. The absorbance was measured at 450 nm (test wavelength) and 650 nm (reference wavelength) using a microplate spectrophotometer (Epoch Multi-Volume Spectrophotometer System, BioTek, VT, USA). The measured absorbances were used to determine cell proliferation according to the following formula:

$$\mathrm{Cell}\;\mathrm{proliferation}\;\left(\%\right)=\frac{{\left({\mathrm{ABS}}_{\mathrm{sample}}-{\mathrm{ABS}}_{\mathrm{blank}}\right)}_{450\mathrm{nm}}-{\left({\mathrm{ABS}}_{\mathrm{sample}}-{\mathrm{ABS}}_{\mathrm{blank}}\right)}_{650\mathrm{nm}}}{{\left({\mathrm{ABS}}_{\mathrm{control}}-{\mathrm{ABS}}_{\mathrm{blank}}\right)}_{450\mathrm{nm}}-{\left({\mathrm{ABS}}_{\mathrm{control}}-{\mathrm{ABS}}_{\mathrm{blank}}\right)}_{650\mathrm{nm}}}\times 100$$

### 4.3. Animal Breeding and Experimental Design

**Figure 8:**
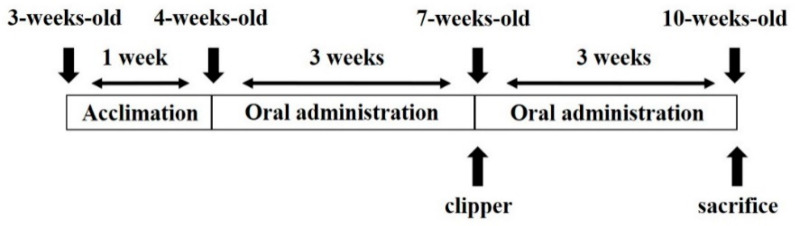
Timeline of experimental treatments and sample collection.

Male C57BL/6 mice (3-weeks-old) purchased from Dae-Han Biolink Co. (Eumsung, Chungbuk, Korea) were allowed to acclimate to laboratory conditions for 1 week. The mice were maintained at 23 ± 2 ℃, a relative humidity of 50 ± 5%, and a 12:12 h light-dark cycle. All animal procedures were performed according to the Guidelines for the Care and Use of Laboratory Animals of the National Institutes of Health, as well as the guidelines established by the Animal Welfare Act. Animals were adapted for one week with free access to pellet food and water until the day of the experiment. After one week of acclimation, the mice were randomly assigned to four experimental groups (*n* = 8/group) and raised for 6 weeks. Thirty-two mice (*n* = 8/group) were randomized into four experimental groups: negative control (NC; distilled water), positive control (PC; 1 mg/kg of finasteride), CP500 (500 mg/kg of CP), and CP1000 (1000 mg/kg of CP). The backs of the mice were shaved with an animal clipper at 7 weeks of age. Distilled water (200 µL), finasteride (1 mg/kg in distilled water), and CP solutions (500 mg/kg and 1000 mg/kg in distilled water) were orally administrated for 6 weeks (Figure 8). Afterward, the mice were anesthetized by intraperitoneal injection of 1.2% avertin to obtain the dorsal skins. The study was approved by the Institutional Animal Care and Use Committee (IACUC) from Chung-Ang University (approval ID: 2021-00042). During the animal experiments, there were no significant differences on the body weight and the food intake among the groups (*p* < 0.05). Moreover, other specific clinical symptoms including death were not shown during the experimental periods.

### 4.4. Evaluation of Hair Regrowth Score and Relative Area for Each Score

To compare the effects of the different treatments on hair regrowth, dorsal skin photographs were taken on the 14th day after hair removal using a digital camera. The hair regrowth score was quantified using the ImageJ program (Broken Symmetry Software, Bethesda, USA) [64]. Hair regrowth was scored from 0 to 3 according to the difference in the dorsal skin color, with 0 indicating no hair growth (pink skin), 1 indicating gray skin (initial hair growth), 2 indicating a darker gray (indicative of more advanced hair growth), and 3 indicating black (fully grown hair). The hair regrowth score was calculated by measuring the ratio of the area of hair regrowth to the total area, and the sum of the area ratio multiplied by the score was averaged for each group [65]. Thus, the minimum value of the hair regrowth score was 0 and the maximum value was 3 (Table 1).

$$\begin{array}{l} Hair\;regrowth\;score\\ =\frac{\left\{\left(\mathrm{area}\;\mathrm{of}\;\mathrm{pink}\right)\times 0+\left(\mathrm{area}\;\mathrm{of}\;\mathrm{gray}\right)\times 1+\left(\mathrm{area}\;\mathrm{of}\;\mathrm{dark}\;\mathrm{gray}\right)\times 2+\left(\mathrm{area}\;\mathrm{of}\;\mathrm{black}\right)\times 3\right\}}{\mathrm{total}\;\mathrm{area}} \end{array}$$

### 4.5. Histological Analysis

The dorsal skin tissues were stained with hematoxylin & eosin (H&E) to characterize the histological changes caused by oral CP administration. The dorsal skin tissues were fixed with 10% neutral formalin and embedded in paraffin blocks using a Tissue Tek Auto TEC Automated Embedder (Sakura, San Francisco, CA, USA). All 5 µm sections were stained with H&E using a slide stainer (Shandon Linistain GLX, Shandon Inc., Pittsburgh, PA, USA). A veterinary pathologist examined the number of hair follicles and their diameter in the tissues (100×). To confirm anagen induction, the pathologist examined the anagen/telogen (A/T) ratio by counting the number of hair follicles in each hair cycle phase (anagen; large and thick, telogen; small and flat) [66,67,68,69].

### 4.6. Reverse-Transcription Quantitative PCR (RT-qPCR) for Cytokine Analysis

The effects of CP treatment on the expression of cytokines involved in hair growth were quantified via RT-qPCR. RNA was extracted from the dorsal skin tissue of each C57BL/6 mouse, after which cDNA was synthesized via reverse transcription. RT-qPCR was conducted using a Piko-real 96 real-time PCR system (Thermo Fisher Scientific Inc., Waltham, MA, USA) using the following protocol: 45 cycles at 95 °C for 15 s, 60 °C for 30 s, and 72 °C for 30 s. Table 2 shows the primer sequences for IGF-1, VEGF, TGF-β1, Krt27, and Gprc5d.

### 4.7. Western Blot Analysis

Dorsal skin tissues were stored at −70 °C. Tissue samples were homogenized in ice-cold RIPA lysis buffer (Millipore, Billerica, MA, USA) for protein extraction. Tissue debris was removed by centrifugation and the resulting supernatants were collected. Protein concentration was then determined using the BCA protein assay (Thermo Scientific, Bartlesville, OK, USA). The protein was separated on 10% SDS polyacrylamide gel and then transferred to PVDF membranes. The membranes were incubated with specific primary antibodies (anti-mouse β-actin, BMP6, anti-rabbit β-catenin, BMP4, and Wnt10b) overnight at 4 °C. After washing, the membranes were allowed to react with diluted horseradish peroxidase-conjugated secondary antibodies (goat anti-rabbit IgG antibody, horse anti-mouse IgG; Cell Signaling Technology Inc., Beverly, MA, USA) for 90 min at room temperature. An enhanced chemiluminescence (ECL) system (SuperSignal, Thermo Scientific, Rockford, IL, USA) was used to visualize antibody–antigen complexes.

### 4.8. Statistical Analyses

Statistical analyses were conducted using SPSS version 25 (SPSS Inc., Chicago, IL, USA). The data are presented as mean ± standard error (SE). Differences between groups were identified using one-way analysis of variance (ANOVA) followed by Duncan’s multiple range test. A *p*-value < 0.05 was considered statistically significant.

## Figures and Tables

**Table 1:** Scores for 4 grades of skin color.

Grade	Skin Color	Score
Grade 1	Pink (no hair)	0
Grade 2	Grey (initial hair growth)	1
Grade 3	Dark grey (visible growth)	2
Grade 4	Black (full grown hair)	3

**Table 2:** Primer pairs used for RT-qPCR.

Target Genes	Primer	Sequence
Ki67	Forward	5′-CCT GCC CGA CCC TAC AAA AT-3′
	Reverse	5′-TCC GCC GTC TTA AGG TAG GA-3′
IGF-1	Forward	5′-TGC TCT TCA GTT CGT GTG-3′
	Reverse	5′-ACA TCT CCA GTC TCC TCA G-3′
VEGF	Forward	5′-TCT TCA AGC CAT CCT GTG TG-3′
	Reverse	5′-GCG AGT CTG TGT TTT TGC AG-3′
TGF-β1	Forward	5′-GGC GGT GCT CGC TTT GTA C-3′
	Reverse	5′-TCC CGA ATG TCT GAC GTA TTG A-3′
Krt27	Forward	5′-TAT GGG CGG TGC TTC TTG TG-3′
	Reverse	5′-TCC AGT GCT TGC ACG TTC TC-3′
Gprc5d	Forward	5′-GGC CCT CAC TTT CTT CGT CTC-3′
	Reverse	5′-GTT CTC ACA TGG GCC ACA GA-3′
GAPDH	Forward	5′-GGG AAG CCC ATC ACC ATC T-3′
	Reverse	5′-CGG CCT CAC CCC ATT TG-3′

## Data Availability

Not applicable.
